# A Review of the Occurrence of Intraventricular Hemorrhage in Preterm Newborns and its Future Neurodevelopmental Consequences

**DOI:** 10.7759/cureus.48968

**Published:** 2023-11-17

**Authors:** Gauri S Pande, Jayant D Vagha

**Affiliations:** 1 Department of Pediatrics, Jawaharlal Nehru Medical College, Datta Meghe Institute of Higher Education and Research, Wardha, IND

**Keywords:** hemoglobin (hb), recombinant human erythropoietin (rhepo), neuroendoscopic lavage, germinal matrix, preterm neonate, intraventricular hemorrhage (ivh)

## Abstract

Intraventricular hemorrhage (IVH) is a type of bleeding that occurs through the germinal matrix and comes through the ependymal cells into the ventricular cavity. It is mostly seen in preterm neonates but can also be seen sometimes in term neonates. Various factors predispose to preterm delivery; it can be spontaneous or medically induced. Spontaneous IVH occurs in cases of intrauterine infections in the mother, and it can be induced in cases of medical emergencies such as preeclampsia and eclampsia. The brain of a preterm newborn is not fully developed as it does not have pericytes and proteins, so it can bleed very quickly, which can cause IVH. Also, the vessels supplying the germinal matrix are immature and highly vascularized. IVH has four grades based on findings detected on cranial ultrasound and MRI. Management includes medical and surgical management; medical management includes phenobarbitone used for seizures and prophylaxis.

Surgical management includes drainage, irrigation, and fibrinolytic therapy (DRIFT), and neuro-endoscopic lavage. IVH causes various short-term and long-term neurodevelopmental consequences. Long-term complications include cerebral palsy and intellectual disability, which hamper the life of the child. It mainly presents with seizures, flaccidity, decerebrate posture, etc. Various preventive measures can be taken to tackle IVH in newborns. First of all, preterm delivery should be avoided, and intrauterine infections in mothers should be treated. The administration of corticosteroids should be done for all preterm deliveries as it helps in the maturation of organs. The administration of magnesium sulfate should be done as it is neuroprotective and reduces cerebral palsy in the future. Delayed cord clamping is to be done to reduce recurrent blood transfusions and decrease the risk of IVH. This article explains the pathogenesis, management, prevention, and future outcomes of IVH.

## Introduction and background

The type of bleeding that occurs in the cavity of the brain's ventricles is called intraventricular hemorrhage (IVH) [1]. It occurs when vessels supplying germinal matrix undergo hemorrhage, and it comes through ependyma in the lateral ventricle [2]. This condition is mostly prevalent in preterm neonates [3], the lower the gestational age, the higher the risk of IVH. It is primarily seen in preterm neonates but also in term neonates [4]. It is more common in infants who are born before 27 weeks of gestation but is also a very prevalent condition seen in preterm neonates who are born before the gestational age of 32 weeks. When these neonates turn into infants, they also experience both short-term and long-term neurological sequelae. The primary attributable causes are genetic factors, germinal matrix moderation, and immaturity of cerebral autoregulation. The extensive use of cranial ultrasonography related the accurate identification of IVH to risk factors associated with the mother and during the conduction of delivery, which resulted in a decrease in the cases of IVH. By the use of MRI, a more accurate apprehension of the location and extent of IVH has been made possible [5]. The increased survival rate of preterm neonates is seen after administering corticosteroids and magnesium sulfate to the mother antenatally [6]. These neonates are born at a very important period of development of the brain, so these children develop both short and long-term neurological, sensory, cognitive, and motor dysfunctions, which depend on the severity of IVH [7]. 

## Review

Methodology

**Figure 1:**
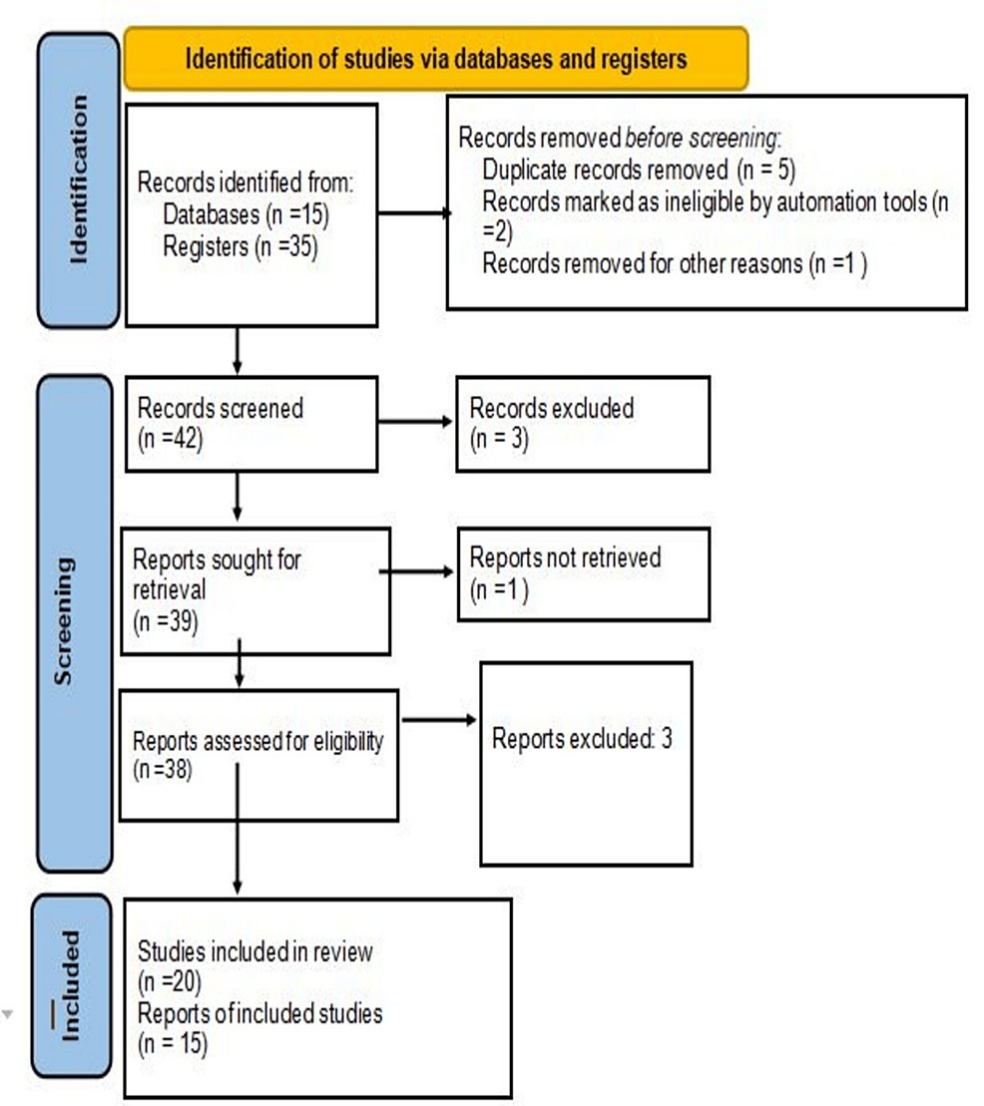
PRISMA flow diagram for literature search

Extensive research was conducted using several electronic databases, PubMed and Google Scholar. The search terms used are intraventricular hemorrhage, preterm newborn, germinal matrix, and neuro-endoscopic lavage recombinant human erythropoietin. A total of 50 articles were studied by the writer, out of which 35 are being referred in this article. The study includes articles which were published from 1990 to the present time. The articles that matched the following criteria were selected in the review: those published in the English language, papers from previous years, and those that are devoted entirely to the occurrence of intraventricular hemorrhage in preterm newborns and its future neurodevelopmental consequences. The research methodology by the preferred reporting items for systematic reviews and meta-analyses (PRISMA) method is shown in Figure 1. 

Preterm Neonates

A neonate who is born before 37 gestational weeks is called a preterm neonate. Depending on gestational age, they can be classified as highly preterm babies who are born before 28 weeks of gestation; very preterm, who are born in 28-32 weeks; moderately preterm, who are born in 32-33 gestational weeks, and late preterm who are born in 34-36 gestational weeks. Preterm deliveries can occur voluntarily through unjustified onset of preterm labor or due to spontaneous rupture of membranes, or they can be artificially induced due to some medical demands.

Risk factors of preterm deliveries: The majority of voluntary and natural preterm births occur due to infections of the uterine cavity, which is followed by smoking by mothers, poor economic status, and multifetal pregnancies. There has been observed an increased usage of assisted reproductive techniques in the present times, which includes in vitro fertilization intracytoplasmic sperm injection which in turn increases the chances of multiple gestation due to administration of ovulation induction drugs, which results in 50% preterm births in all the total birth by IVF. The risk of development of various diseases is seen in preterm babies, such as heart-related (patent ductus arteriosus), respiratory (respiratory distress syndrome and bronchopulmonary dysplasia), and neurological conditions, which include IVH. These pathologies are responsible for the increased risk of mortality in preterm infants [8]. 

Neurological Complications of Preterm Neonate

Germinal matrix-intraventricular hemorrhage (GM-IVH) is a foremost issue of prematurity and is inversely proportional to the age of gestation and birth weight. The germinal matrix is a place in the ventricular cavity of the brain having an immature capillary bed and an extremely high blood supply and where multiplication rates of the active cell are higher. All preterm babies who are delivered before 32 gestational weeks are advised to undergo screening with the use of cranial USG. It is also recommended in all high-risk newborns who are delivered after 32 weeks of gestation but are not clinically stable and have abnormal neurologic findings. But cranial USG does not show white matter injury; so, to accurately diagnose it, the gold standard is MRI. However, MRI is not easy for most babies as they are unstable for transportation in the early period, and imaging takes longer [9].

Epidemiology: The global occurrence rates of IVH range from 3.70 to 44.68%. According to a study, the global incidence is 36.2%, with grade III and grade IV, which are called severe grades, present in 7.1% of them. The general frequency of IVH grades I, II, III, and IV in preterm infants is 17.0%, 12.1%, 3.3%, and 3.8%, respectively. Around 50% of IVH occurs by day one of birth, increasing to 90% by day three. As the gestational age advances, an increase in a week in gestational age with a weight of 1000g or less decreases the risk of severe IVH by 19%. Till the gestational age of 32 weeks, when the age increases by one week, there is a 3.5% decline in IVH. In newborns of gestational age between 22 and 28 weeks, an overall prevalence of 32% exists. More than 31 gestational weeks and a weight of more than 1500g rarely results in grade III or grade IV IVH. Based on weight, in cases of newborns less than 1000g, the incidence is 45%, and in less than 1500g, it is 25 to 30% [10].

Etiology

The primary causative mechanism of IVH is the weakness of the germinal matrix vessels and the immature cerebral autoregulation in newborns. The configuration of vessels of a germinal matrix of newborns is different than that of other areas of the cerebral cortex. It is so because there is an increase in metabolic demand by precursor cells due to their rapid turnover in this region. Germinal matrix blood vessels are higher in density and surface area than other cortex areas. Also, regarding vascular morphology, the germinal matrix vessels are more spherical than cortical vessels, which are more flat; all these factors result in vascular immaturity of the germinal matrix vessel. Trauma to the white matter of the brain surrounding the germinal matrix occurs from IVH due to the expansion of the ventricles, which presses over it. There occurs weakening of the ependymal lining, so direct injury can also happen to white matter. When trauma occurs, it causes stretching of the ependymal lining, causing its rupture, which exposes the white matter to blood and other reactive components of cells. The primary risk factor is the transportation of newborns after delivery. Other neonatal factors also contribute, such as mechanical ventilation, increased carbon dioxide concentration in blood, spontaneous pneumothorax, and recurrent endotracheal intubations. The frequent alteration in blood pressure of neonates also contributes to IVH [10]. 

Anatomy of IVH

During the maturation of the nervous system, the site of the multiplication of neurons of the cortex and glial precursors is situated in the germinal matrix and the adjacent ventricular germinal matrix. Microglia and microneurons are formed in the late second and third trimesters of pregnancy in these regions by mitosis. As these areas develop and fully mature after 34 gestational weeks, IVH is predominantly seen in the preterm newborn. IVH originates in the periventricular matrix zone between the caudate nucleus and the thalamus at the level of, or slightly posterior to, the foramen of Monroe. At 28 weeks of gestation, IVH predominantly occurs in the germinal matrix at the head of the caudate nucleus at the level of the foramen of Monroe. But in cases of the full-term neonate, it arises from the choroid plexus, and in cases of a preterm infant, very few instances of IVH arise from this site. The capillaries of the germinal matrix are composed of large, immature vessels with loss of pericytes and protein and structures supporting glia. Grunnet, in his study, has demonstrated that the lumen of the germinal matrix vessels are larger than those of cortical vessels of neonates of the same gestational age and has thus hypothesized that, because of their larger diameter, immense pressure can act on their walls, thereby increasing their vulnerability to burst [11].

Pathogenesis

The pathologic process of IVH is diverse and complicated and is contributed by multiple factors, mainly the fragile blood vessels of the germinal matrix. Due to low mean arterial pressure across the germinal vessels, there occur alterations in blood flow to the brain, which damages the cerebral autoregulation in preterm newborns, rendering them clinically unstable. All these factors play a combined role in increasing the risk of rupture of vessels, leading to hemorrhage in the area of the germinal matrix, which can also extend up to the adjacent lateral ventricle. When there is fetal hypoxia, it initiates the process of erythropoiesis by increasing the release of growth factors such as vascular endothelial growth factor and angiopoietin 2, which promotes neovascularization [12]. This, in turn, will lead to the production of weak and fragile new vessels that lack pericytes (pericytes are cells present along the walls of capillaries) [13], which have low levels of fibronectin in the immature basal lamina, and also astrocytes that are deficient in glial fibrillary acidic protein.

If a neonate is suffering from underlying platelet or coagulation disorder, it accelerates the process of hemorrhage. When a hematoma causes occlusion of the vein, there is an impairment of perfusion in the white matter, which causes hemorrhagic parenchymal infarction [12]. The germinal matrix blood vessels are located in sub-ependymal regions. Capillary networks rapidly supply them, and they are not supported by muscle or collagen, so they can undergo sudden changes in hemodynamics, making them more susceptible to rupture [14]. In preterm neonates, the risk of developing severe IVH has increased if there is hemodynamically significant patent ductus arteriosus present. Polymorphisms in the gene coding for the metabolism of Vitamin K (vitamin K epoxide reductase complex 1) and transport of Vitamin K (APOE2 and APOE4) have an impact on the possibility of IVH development in preterm neonates [15]. The brains of preterm neonates are more vulnerable to reactive oxygen species or free radicals than mature brains because of the immaturity of the antioxidant enzyme system, which is used to detoxify them [16].

Risk factors

Prenatal risk factors include infection of membranes (chorion and amnion), the mother having hemorrhagic disorders, and prothrombin mutation such as factor 5 leaden. Some factors also play a protective effect on IVH; they are preeclampsia in the mother (a condition of increased blood pressure), in Caesarean section when delayed cord clamping is done, etc. It is a widely accepted fact now that the administration of corticosteroids to mothers antenatally before delivery reduces mortality and morbidity of preterm neonates [17]. In the first few hours of life, there is the presence of a hypercoagulable state, so it predisposes the neonate to the risk of IVH [18].

Clinical features

According to a study conducted, the most frequent clinical symptom which was observed is seizure followed by poor feeding [19]. In about two-thirds of newborns with IVH, focal onset and generalized seizure are observed mainly in the first 48 hours after birth. Other features include flaccidity, incapacity in the movements of extraocular muscles, and abnormal respiratory movements; the child can suffer from respiratory distress syndrome (RDS), and the neurological symptoms include irritability, crying, possibly fontanelles being full, restless movements, and ultimately, coma, and opisthotonos posturing. There will be several metabolic abnormalities, which include hypoglycemia, fever, hyperglycemia, and hypothermia. On examination, there will be hypotonia in the lower limbs and in neck flexion. There will be head lag and brisk or absent reflexes [20]. With extensive hemorrhage, a catastrophic deterioration occurs with stupor, decerebrate posturing, generalized tonic seizures, and hypotonia.

Investigation: Cranial ultrasound (CUS) gives an accurate diagnosis of IVH as well as tells about the progression of the disease. It should be a rapid procedure and gentle to decrease stress among the neonates [21]. Earlier, only CUS was used to diagnose, but nowadays, MRI is also used to get an accurate diagnosis [22]. CUS has been in use since the late 70s for diagnosing IVH, but now MRI is being widely used. CUS is readily available and commonly used in investigations in neonatal intensive care units [23].

Management

Management can be done in two ways: medical management and surgical management.

Medical Management

1) For preventing IVH - Phenobarbitone: Its mechanism of action is to reduce hemodynamic stress on the germinal matrix vessel wall. In a 2013 Cochrane review of 12 controlled trials, it was seen that phenobarbitone does not decrease the risk of IVH. It was concluded that phenobarbitone cannot be used for the prevention of IVH in preterm newborns [8].

2) For stepping down the progress of IVH - Ethamsylate (diethylammonium 1,4-dihydroxy-3-benzene sulfonate): It provides basement membrane stability and reduces the bleeding time and loss of blood from the wound. This is why it is advised to be used in preterm neonates. According to a study [8], it also decreases the possibility of grade III and grade IV GM-IVH. Hypotension is the side effect of ethamsylate, but it is safe for newborns.

3) For enhancing the neurological results of IVH - Recombinant human erythropoietin (EPO): It enhances the process of erythropoiesis; therefore, it helps reduce the frequency of blood transfusion. When high-dose erythropoietin was given in neonates of less than 32 weeks gestational age, it resulted in an increase in packed cell volume, reticulocyte index, leucocyte count, and platelet count [8]. According to a study, when preterm neonates have been treated with erythropoietin, it has improved the future outcome of IVH by minimizing the need for blood transfusion [24]. Vitamin E (tocopherol) is a potential antioxidant and hunts reactive oxygen species, and it protects the vascular endothelial cells of the germinal matrix from trauma. It reduces the occurrence of IVH but escalates the frequency of sepsis in neonates [8].

Surgical Management 

1) Drainage, irrigation, and fibrinolytic therapy (DRIFT): According to a study, it improves cognitive function. However, it is not a standard treatment for IVH. In this, we remove the hematoma and results of blood clot lysis after fibrinolytic therapy by administering intracerebroventricular infusions of recombinant tissue plasminogen activator. It improves the future neurological outcomes of IVH [25]. 

2) Neuro-endoscopic lavage: A study has shown that neuro-endoscopic lavage in newborns with IVH had fewer shunts, fewer infections, and fewer rates of hydrocephalus than those treated with conventional treatment [26].

Future outcomes of IVH

Moderate to severe type of hemorrhage dilates the ventricles, so it compresses the surrounding white matter around the ventricles and damages it. IVH has very long-term neurodevelopmental effects, so in children of age groups 5-10 years, it showed an increased occurrence of cerebral palsy [25]; there can be quadri-paresis, diparesis, or hemiparesis [27]. There can be cognitive impairment in them as compared to those who do not have IVH at birth. The incidence of other psychiatric disorders, such as depression and obsessive-compulsive neurosis, was higher in these children having IVH. The prevalence of attention deficit hyperactivity disorder (ADHD) is increased with IVH [25]. Sherlock et al., in their study from the early 2000s, concluded that at eight years of age, children who had IVH of grades I and II have a similar cognitive function as that of children of the same age with no IVH [28]. Besides white matter, it also causes injury to grey matter as it interferes with neuron and cortex formation and reduces the number of neurons and the volume of the cortex [29]. IVH has also been seen in association with visual impairment and hearing loss [30]. Compression of periventricular tissue will result from dilatation of the ventricle, which leads to reversible but decreased arterial blood flow, which results in excitotoxicity and causes white matter damage [31].

Prevention

A combined approach is needed to protect preterm babies using antenatal, intranatal, postnatal, and NICU approaches [32]. Preterm birth unless medically needed (as in cases of severe preeclampsia with fetal growth restriction) can be avoided. Antenatal progesterone should be given to expectant mothers at 34 weeks who have a previous history of preterm birth or have a short cervical length. Other steps can be taken, such as avoiding tobacco and smoking during pregnancy, doing cervical cerclage if cervical incompetence is present, and the tocolytics can be given to prevent preterm labor. Prenatal corticosteroids should be given to expectant mothers between 24 to 34 weeks whose preterm delivery is inevitable. It reduces the occurrence of IVH and white matter injury in the preterm newborn by administering betamethasone or dexamethasone to the mother. It provides stability to germinal matrix vasculature [12]. 

Corticosteroids show the effects of vasoconstriction on cerebral blood flow and protect the fetus against IVH. [33]. Prenatal administration of magnesium sulfate to the mother provides neuroprotection against cerebral palsy. Many studies suggested that IV administration of indomethacin after birth protects from IVH by stimulating basement membrane deposition in the germinal matrix vessels [12]. Intrauterine infections in mother play a significant role in preterm delivery and also increases the risk of IVH in the neonate, so special measures should be taken to prevent them [34]. Postnatally delayed cord clamping (DCC) is done in all term and preterm infants except those who need immediate resuscitation measures after birth [35]. DCC increases the hematocrit, blood flow of the inferior vena cava, output from the right ventricle, increases blood pressure and temperature, lessens the rates of resuscitation in the delivery room, and also reduces the need for early blood transfusion. DCC is beneficial in the prevention of GM-IVH [12].

A summary of the various studies included in this review is provided in Table 1.

**Table 1:** Summary table of the studies included in the review IVH: intraventricular hemorrhage; VEGF: vascular endothelial growth factor; RDS: respiratory distress syndrome; DRIFT: drainage, irrigation, and fibrinolytic therapy

Authors	Year	Country	Findings
Hambleton G, et al. [1]	1976	UK	Intraventricular hemorrhage is a type of bleeding that occurs in the ventricular cavity of the brain.
Siffel C, et al. [2]	2021	USA	The cause of hemorrhage is the vessels supplying the germinal matrix of the lateral ventricle.
Trounce JQ, et al. [3]	1986	UK	This condition is primarily encountered in neonates who are born before the completion of the term.
Shah V, et al. [4]	2022	Canada	The prevalence of IVH is indirectly proportional to that of gestational age.
Reddy PA, et al. [5]	2023	India	The primary responsible factors are genetic factors and cerebral dysautoregulation. By the use of MRI, the location and extent of hemorrhage can be easily found.
Gilard V, et al. [6]	2020	France	By administration of corticosteroids and magnesium sulfate to the mother antenatally, the prevalence of IVH is decreased.
Radmila MM, et al. [7]	2021	Serbia	These children develop future cognitive and neurological sequelae.
Navarro IA, et al. [8]	2020	Spain	The preterm neonate is classified as late preterm, moderately preterm, and late preterm. There are various risk factors which are associated with preterm delivery.
Özek E, et al. [9]	2020	Turkey	The gold standard for diagnosis is MRI, but neonates born before 32 weeks should undergo screening by cranial USG.
Starr R, et al. [10]	2023	USA	Pathogenesis is weakened germinal vessels and defects in cerebral autoregulation.
Vohr B, et al [11]	1996	USA	In the developing nervous system, the site of the multiplication of neurons of the cortex and glial precursors is situated in the germinal matrix and the adjacent ventricular germinal zone.
Egesa WI, et al [12]	2021	Uganda	Due to fetal hypoxia, there occurs release of VEGF, which causes neovascularization.
Rodríguez MH, et al [13]	2017	Mexico	New blood vessels are formed, which are fragile and lack pericytes.
Tortorolo G, et al [14]	1999	Italy	Capillaries richly supply germinal vessels and are situated in the ependymal region.
Tsao PC, et al. [15]	2023	Taiwan	Patent ductus arteriosus has an increased risk of IVH.
McCrea JH, et al. [16]	2008	USA	Immaturity of antioxidant mechanism in the preterm neonate, so more vulnerable to oxidative stress.
MOTLAGH AJ, et al [17]	2021	Iran	Risk factors include infection of chorion and amnion, RDS of neonate.
Bruschettini M, et al [18]	2016	Italy	Hypercoagulable state in the early few hours of life predisposes to IVH.
Spagnoli C, et al [19]	2018	Italy	The most frequent clinical symptom is seizure followed by frequent feeding.
Afsharkhas L, et al. [20]	2015	Iran	Other features include flaccidity and loss of reflexes.
Parodi A, et al. [21]	2020	Italy	Cranial ultrasound gives accurate diagnosis and progression of disease.
Dunbar MJ, et al [22]	2021	Canada	MRI is now used these days/
Brouwer AJ, et al [23]	2014	Netherlands	In neonatal ICU, cranial ultrasound still is used.
Wellmann S, et al. [24]	2022	Germany	For treatment, phenobarbitone, ethamsylate, and recombinant human erythropoietin are used.
Ballabh P, et al [25]	2021	USA	A surgery is done called DRIFT.
Kuo MF, et al. [26]	2020	Taiwan	Neuroendoscopic lavage is used, which has fewer infections.
O’Shea TM, et al. [27]	2012	USA	In the future, the child can suffer from quadriparesis, diaphoresis, or hemiparesis.
Legge N’ et al [28]	2022	Australia	The child can also suffer from cognitive impairment, depression, psychosis, and obsessive-compulsive disorder.
Sharma DR, et al. [29]	2022	USA	Injury to grey matter also occurs.
Bueno GGG, et al [30]	2019	Brazil	It can also lead to visual impairment and hearing loss.
Adler I, et al. [31]	2010	USA	Compression of periventricular tissue also causes white matter damage.
Ment LR, et al. [32]	2015	USA	For prevention, the child needs to be provided prenatal, intranasal, and postnatal care.
McGoldrick E, et al [33]	2020	UK	Corticosteroids to mother is useful.
Huang J, et al. [34]	2019	China	Magnesium sulfate is given to the mother and delayed cord clamping is done to the children.
Jasani B, et al [35]	2021	Canada	Delayed cord clamping is done in all neonates except those who need resuscitation.

## Conclusions

Hence, to conclude, IVH is a complication of preterm delivery due to the immature development of the brain. The vasculature supplying the germinal matrix is immature and weak and does not have a pericyte, so it is prone to hemorrhage. There are four grades to it; grades III and IV are severe grades of IVH. Clinical features include seizures, decerebrate posture, flaccidity, and several other features; it is diagnosed by doing CSU and MRI. Medical interventions include phenobarbitone, ethamsylate, and recombinant human erythropoietin (it decreases the frequency of blood transfusions), decreasing the frequency of IVH; vitamin E can also be given. Various preventive measures need to be taken to prevent IVH in preterm newborns because it causes various short-term and long-term sequelae. Cerebral palsy and intellectual disability are dreadful complications of IVH; they hamper the life of a child, so they should be managed in a very skillful way. In cerebral palsy, there occurs paralysis of limbs, so good preventive measures need to be taken. First of all, preterm delivery should be avoided, and intrauterine infections in mothers should be treated; administration of corticosteroids for all preterm deliveries helps in the maturation of organs. Administration of magnesium sulfate reduces cerebral palsy in the future as it is neuroprotective. DCC provides preload to the right side of the heart by improving flow to the inferior vena cava; it stabilizes blood pressure and prevents sudden changes in it that could have led to IVH and, hence, decreases the risk of IVH.
